# Extensively Drug-Resistant *Klebsiella pneumoniae* Causing Nosocomial Bloodstream Infections in China: Molecular Investigation of Antibiotic Resistance Determinants, Informing Therapy, and Clinical Outcomes

**DOI:** 10.3389/fmicb.2017.01230

**Published:** 2017-06-30

**Authors:** Wenzi Bi, Haiyang Liu, Rhys A. Dunstan, Bin Li, Von Vergel L. Torres, Jianming Cao, Lijiang Chen, Jonathan J. Wilksch, Richard A. Strugnell, Trevor Lithgow, Tieli Zhou

**Affiliations:** ^1^Department of Clinical Laboratory, The First Affiliated Hospital of Wenzhou Medical UniversityWenzhou, China; ^2^School of Laboratory Medicine and Life Science, Wenzhou Medical UniversityWenzhou, China; ^3^Infection and Immunity Program, Biomedicine Discovery Institute and Department of Microbiology, Monash UniversityMelbourne, VIC, Australia; ^4^Department of Microbiology and Immunology, The Peter Doherty Institute, The University of MelbourneParkville, VIC, Australia

**Keywords:** antimicrobial resistance determinants, bacteraemia, clinical outcomes, *Klebsiella pneumoniae*, XDR

## Abstract

The rise in diversity of antimicrobial resistance phenotypes seen in *Klebsiella pneumoniae* is becoming a serious antibiotic management problem. We sought to investigate the molecular characteristics and clinical implications of extensively drug-resistant (XDR) *K. pneumoniae* isolated from different nosocomial bloodstream infections (BSIs) patients from July 2013 to November 2015. Even in combination treatment, meropenem did not protect against mortality of BSIs patients (*P* = 0.015). In contrast, tigecycline in combination with other antimicrobial agents significantly protected against mortality (*P* = 0.016). Antimicrobial susceptibility tests, molecular detection of antibiotic resistance determinants, conjugation experiments, multilocus sequence typing (MLST), S1-PFGE, Southern blot, SDS-PAGE, immunoblot analysis, and pulsed-field gel electrophoresis (PFGE) were used to characterize these isolates. These XDR *K. pneumoniae* strains were resistant to conventional antimicrobials except tigecycline and polymyxin B and co-harbored diverse resistance determinants. *rmtB, bla*_KPC−2_ as well as *bla*_CTX−M−9_ were located on a transferable plasmid of ~54.2 kb and the most predominant replicon type was IncF. 23 of the 35 isolates belonging the predominant clone were found to incorporate the globally-disseminated sequence type ST11, but others including a unique, previously undiscovered lineage ST2281 (allelic profile: 4-1-1-22-7-4-35) were also found and characterized. The porins OmpK35 and OmpK36 were deficient in two carbapenemase-negative carbapenem-resistant strains, suggesting decreased drug uptake as a mechanism for carbapenem resistance. This study highlights the importance of tracking hospital acquired infections, monitoring modes of antibiotic resistance to improve health outcomes of BSIs patients and to highlight the problems of XDR *K. pneumoniae* dissemination in healthcare settings.

## Introduction

The Gram-negative bacterium *Klebsiella pneumoniae* is widely distributed in the environment and increasingly reported as a cause of invasive infections in healthcare settings, particulary in immunocompromised patients (Bagley, 1985; Lee et al., 2016; Paczosa and Mecsas, 2016; Wyres and Holt, 2016). Antimicrobial resistance in *K. pneumonia*e is increasing, particularly beta-lactamases and carbapenemases having been well-characterized as increasing the infection threat (Mathers et al., 2015; Campos et al., 2016; Lee et al., 2016). This seriously antibiotic management problem is now frequently seeing both nosocomial and community associated infections. Infections caused by extensively drug-resistant (XDR) *K. pneumoniae*, such as pneumonia, urinary tract infections, and bloodstream infections (BSIs) (Bagley, 1985; Girometti et al., 2014; Paczosa and Mecsas, 2016), have been closely related to increased morbidity, mortality, long hospital stay, and high healthcare costs (Giske et al., 2008; Bush et al., 2011).

Antimicrobial resistance is a global problem, and *K. pneumoniae* is recognized as a major pathogen of hospital-acquired infections. In the past several years, Chinese clinicians have witnessed a remarkable increase in the drug resistance rate of *K. pneumoniae* strains isolated from clinical settings. For example, carbapenemase-producing *K. pneumoniae* was first identified in China in 2007 and by 2013 a carbapenemase-resistance cassette was carried by 13.4% of *K. pneumoniae* isolated from hospital patients (Qi et al., 2011; Hu et al., 2016). Now, a substantial portion of hospitalized patients are colonized by these pathogens, causing outbreaks of nosocomial infections in various regions across the country (Paczosa and Mecsas, 2016). *K. pneumoniae* is now seen as one of the major pathogenic bacteria of BSIs, accounting for 11.3% between 2011 and 2012 in China (Lv et al., 2014). The dissemination of XDR *K. pneumoniae* is now causing difficult-to-treat infections worldwide, bringing with its tremendous challenges to the clinical therapeutic options (Lim et al., 2015). Although carbapenems possess good antibacterial activity to Gram-negative bacteria, the rates of carbapenem resistance among *K. pneumoniae* escalated from 0.7% in 2006 to 10% in 2013 (Hu et al., 2016). The availability of alternative, effective antimicrobial agents is limited (Tang et al., 2016).

Several mechanisms are known to mediate antibiotic resistance to commonly used antimicrobial agents, including extended-spectrum β-lactamases (ESBLs) and carbapenemases, as well as plasmid-mediated quinolone resistance (PMQR) genes, aminoglycoside-modifying enzymes (AMEs), and 16S rRNA methyltransferase (16S-RMTase) (Hu et al., 2014; Findlay et al., 2015; Buruk et al., 2016). The current study focused on pinpointing the antibiotic resistance determinants of XDR *K. pneumoniae* isolated from nosocomial BSIs patients and factors closely related to clinical outcome, with emphasis on determining appropriate antimicrobial drug therapy.

## Materials and methods

### Clinical data collection

From July 2013 to November 2015, a total of 131 BSIs patients hospitalized in the First Affiliated Hospital of Wenzhou Medical University were shown to carry *K. pneumoniae*. Among these, 35 patients were defined as XDR *K. pneumoniae* BSIs patients if their blood cultures were positive for XDR *K. pneumoniae* and with clinical signs of systemic inflammatory response syndrome. BSIs patients were further divided into two groups based on clinical outcome of antimicrobial treatment: death group (*n* = 14) and survivor group (*n* = 21). Medical records and clinical data (patient age, gender, ICU length of stay, reasons for ICU admission, intensive care procedures, comorbidities, medical care measures before BSIs, antibiotic administration, microbiological data, and outcomes) were collected and analyzed and are presented in Table 1.

**Table 1:** Clinical characteristics of nosocomial BSIs patients.

**Characteristics of BSIs patients variable**	**All patients (*n* = 35)**	**BSIs patients**		***P***
		**Death group (*n* = 14)**	**Survivor group (*n* = 21)**	
Age (years), median (IOR)	60 (14–90)	65 (14–90)	59 (16–83)	
Male	28 (80.0)	11 (31.4)	17 (48.6)	1
**Type of ICU admission**				
Direct	13 (37.1)	6 (17.1)	7 (20.0)	0.724
Transfer	8 (22.9)	5 (14.3)	3 (8.6)	0.221
**Reason for ICU admission**				
Respiratory failure	6 (17.1)	1 (2.8)	5 (14.3)	0.366
Shock	4 (11.4)	3 (8.6)	1 (2.8)	0.279
Coma	6 (17.1)	5 (14.3)	1 (2.8)	0.028
**ICU intensive care procedures**				
Invasive mechanical ventilation	16 (45.8)	8 (22.9)	8 (22.9)	0.317
Central venous catheter	18 (51.4)	8 (22.9)	10 (28.5)	0.733
Urinary catheter	12 (34.3)	8 (22.9)	4 (11.4)	0.031
**Pre-infection health care interventions**				
Surgery	8 (22.9)	5 (14.3)	3 (8.6)	0.221
Dialysis	6 (17.1)	2 (5.7)	4 (11.4)	1
Mechanical ventilation	17 (48.6)	8 (22.9)	9 (25.7)	0.5
**Indwelling invasive devices**				
Central venous catheter	28 (80.0)	13 (37.1)	15 (42.9)	0.203
Urinary catheter	20 (57.2)	12 (34.3)	8 (22.9)	0.007
**Treatments administered**				
Corticosteroids	11 (31.4)	4 (11.4)	7 (20.0)	1
Chemotherapy, radiotherapy	6 (17.1)	1 (2.8)	5 (14.3)	0.366
**Comorbidities**				
Intracranial disease	7 (20.0)	6 (17.1)	1 (2.8)	0.01
Respiratory disease	27 (77.2)	10 (28.6)	17 (48.6)	0.685
Cardiovascular disease	11 (31.4)	4 (11.4)	7 (20.0)	1
Tumor, leukemia, and lymphoma	5 (14.2)	1 (2.8)	4 (11.4)	0.627
Diabetes	8 (22.9)	3 (8.6)	5 (14.3)	1
Trauma	4 (11.4)	2 (5.7)	2 (5.7)	1
Kidney disease	11 (31.4)	4 (11.4)	7 (20.0)	1
**Combination drug therapy**				
Combination including tigecycline (50 mg/12 h)	14 (40.0)	2 (5.7)	12 (34.3)	0.016
Combination including meropenem (1 g/8 h)	9 (25.7)	7 (20.0)	2 (5.7)	0.015
**Length of stay**				
ICU length of stay, days	16 (1–58)	15 (1–58)	17 (3–57)	
Hospital length of stay, days	30 (1–86)	40 (1–60)	16 (6–86)	

*Data are presented as number (%) or median [IQR]*.

All of the investigation protocols in this study were approved by The Ethics Committee of The First Affiliated Hospital of Wenzhou Medical University. Informed consent was waived because this retrospective study with retrospective observational nature mainly focused on bacteria and did no interventions to patients.

### Bacterial isolates identification and antimicrobial susceptibility profiling

This retrospective study was conducted at the First Affiliated Hospital of Wenzhou Medical University, China. Based on the standardized international definition for XDR described by Li et al. (2012), 35 non-repetitive *K. pneumoniae* strains, isolated from nosocomial BSIs patients that were found to be susceptible to two or fewer antimicrobial categories, were collected. Initially, bacterial identification and antimicrobial susceptibility tests were conducted by the Vitek2 system (BioMèrieux, France). Then, the isolates were stored in frozen condition at −80°C with 30% glycerol. Subsequently, minimum inhibitory concentrations (MICs) of tigecycline and polymyxin B were determined by broth dilution method and interpreted by the recommendation of the European Committee on Antimicrobial Susceptibility Testing (EUCAST, 2015). *Escherichia coli* ATCC 25922 served as the control strain for susceptibility testing.

### Molecular detection of antibiotic resistance determinants

The presence of resistant mechanisms, including ESBLs genes (*bla*_CTX−M−1_, *bla*_CTX−M−9_, *bla*_TEM_, *bla*_SHV_, *bla*_VEB_, and *bla*_PER_), AmpC genes (*bla*_CMY_, *bla*_FOX_, *bla*_MOX_, *bla*_DHA_), carbapenemase genes (*bla*_KPC_, *bla*_SPM_, *bla*_IMP_, *bla*_VIM_, *bla*_GES_, *bla*_NDM_, *bla*_OXA−23_, *bla*_OXA−48_), PMQR genes [*qnrA, qnrB, qnrC, qnrD, qnrS, qepA, aac(6*′*)-Ib-cr, oqxA, oqxB, gyrA, parC*], AMEs [AAC(6′)-Ib, APH(3′)-Ia, AAC(3)-IV, ANT(2″)-Ia], and 16S-RMTase genes (*armA, rmtA, rmtB, rmtC, rmtD, rmtE*) were investigated by PCR and sequencing. For each isolate, DNA was extracted from fresh bacterial colonies using an AxyPrep Bacterial Genomic DNA Miniprep kit (Axygen Scientific, Union city, CA, USA). PCR assays were performed on a Veriti 96-well Thermal Cycler (Bio-Rad, USA) using specific primers, corresponding to related studies (Jacoby, 2009; Yu et al., 2009; Li et al., 2012; Ramirez and Tolmasky, 2013). Primer sequences are available on request. BLAST was utilized to align drug-resistance gene nucleotide sequences (http://blast.ncbi.nlm.nih.gov/Blast.cgi).

### Conjugation experiments

To determine the transferability of resistance determinants, Luria-Bertani (LB) mating experiments were implemented. Sodium azide-resistant *E. coli* J53 served as the recipient strain (Yi et al., 2012). Transconjugants were selected on Mueller-Hinton agar plates containing sodium azide (100 mg/L) and ertapenem (0.5 mg/L). The resistance genes successfully transferred from the donor strains were verified by PCR. MICs of antibiotics for the transconjugants were compared to donors and *E. coli* J53 to further confirm the transferable resistance genes.

### Plasmid analysis and southern blot

Major plasmid incompatibility groups: F, FIA, FIB, FIC, HI1, HI2, I1, L/M, N, P, W, T, X, Y, K, A/C, B/O, FII, FrepB, were detected by a PCR-based replicon typing (PBRT) scheme (Carattoli et al., 2005). S1 nuclease converted supercoiled plasmids into full-length linear molecules, and S1-PFGE can be used to screen for megaplasmids simultaneously (Barton et al., 1995). Total DNA from the *K. pneumoniae* donor strains and *E. coli* transconjugants were isolated using an Axyprep Bacterial Genomic DNA Miniprep kit, digested with S1 nuclease (Takara Bio, Inc.) and analyzed in a CHEF-Mapper XA PFGE system (Bio-Rad). The gel was then subject to Southern blot analysis, after transfer to a positively charged nylon membrane (Roche Diagnostics, Branford, USA) by the capillary method. The membrane was subject to hybridization with labeled *bla*_KPC−2_, *rmtB, bla*_CTX−M−9_ probes according to the instructions of Detection Starter Kit II (Roche, Sant Cugat del Vallès, Spain). The plasmids of *Salmonella* H9812 served as size markers (Zhou et al., 2015).

### Outer membrane proteins analysis

A crude outer membrane protein (OMP) fraction was isolated by sonication, and OMPs were separated by sodium dodecyl sulfate polyacrylamide gel electrophoresis (12% SDS-PAGE), and OmpK35 and OmpK36 were detected by immunoblotting (serum dilution 1:20,000). *K. pneumoniae* ATCC 13883 served as a control strain for OMP profiling (Webb et al., 2012; Zhou et al., 2015). For outer membrane purification, crude membranes were isolated from *K. pneumoniae* ATCC 13883 and subjected to sucrose density fractionation. Membrane fractions were then subject to analysis by SDS-PAGE and Coomassie blue staining. Alternatively, fractions were analyzed by immunoblotting to determine the localization of inner (F_1_β) and outer (BamB) membrane proteins markers (Clements et al., 2009; Zhou et al., 2015).

### Bacterial clonal relatedness

Clonal relatedness for the 35 *K. pneumoniae* strains was analyzed by pulsed-field gel electrophoresis (PFGE). Genomic DNA was extracted using an AxyPrep Bacterial Genomic DNA Miniprep kit, subjected to complete digestion with the restriction endonuclease *XbaI* (Takara Bio, Dalian, China), and the diagnostic DNA fragments then separated in a PFGE CHEF-Mapper XA system (Bio-Rad) using 0.5 × Tris-borate-EDTA buffer at 120 V for 19 h, with pulse times ranging from 5 to 35 s. DNA fragments were stained with Gel Red (Biotium, USA) and analyzed using Quality one software (Bio-Rad). The DNA fingerprint patterns were analyzed according to the criteria proposed by Tenover et al. (Hu et al., 2013) and strains with more than 80% similarity were regarded as the same clone. Molecular typing of XDR *K. pneumoniae* strains was performed according to the protocol described on the Institut Pasteur *K. pneumoniae* MLST website (http://bigsdb.web.pasteur.fr/klebsiella/klebsiella.html). The sequences of seven housekeeping genes (i.e., *gapA, infB, mdh, pgi, phoE, rpoB*, and *tonB*) were amplified and sequence types (STs) were assigned by the MLST database according to Diancourt et al. (2005). The novel STs were submitted to the MLST database.

### Statistical analysis

Categorical variables were compared with the chi-square test using SPSS software (version 17.0). Calculated *p*-values of < 0.05 were considered to be statistically significant.

## Results

### Clinical characteristics of BSIs patients

There is a growing trend of mortality in hospital patients caused by infections with *K. pneumoniae* (Paczosa and Mecsas, 2016). A total of 35 nosocomial BSIs patients (age 14–90 years; male: female 4:1) were included in the present study (Table 1). These patients had been admitted in the intensive care unit (60.0%), hematology department (28.6%), and neurosurgery department (11.4%), data not shown. The BSIs patients were primarily elderly, suffered severe comorbidities and had been submitted to invasive procedures, such as central venous catheterization and urinary catheterization. As documented in Table 1, the BSIs resulted in lengthy ICU stays (1–58 days; median 16 days), within long overall hospital length of stay (1–86 days; median 30 days). The mortality rate attributed to BSIs caused by XDR *K. pneumoniae* was 40.0%, i.e., 14 of the 35 patients died from the BSI. Two groups of BSI patients had an increased risk of death, those that received urinary catheterization (34.3% died vs. 22.9% survived, *P* = 0.007), and patients with intracranial disease (17.1% died vs. 2.8% survived, *P* = 0.01). In terms of their treatment, combination therapy including meropenem proved to be ineffective against mortality of BSIs patients caused by XDR *K. pneumoniae* (*P* = 0.015), but tigecycline in combination with other antimicrobial agents was significantly effective and reduced the risk of death (*P* = 0.016). Furthermore, nine patients of the survivor group were treated with fosfomycin combined with more than three other antibiotics.

### Genotypes and clonal relatedness of XDR *K. pneumoniae*

**Figure 1:**
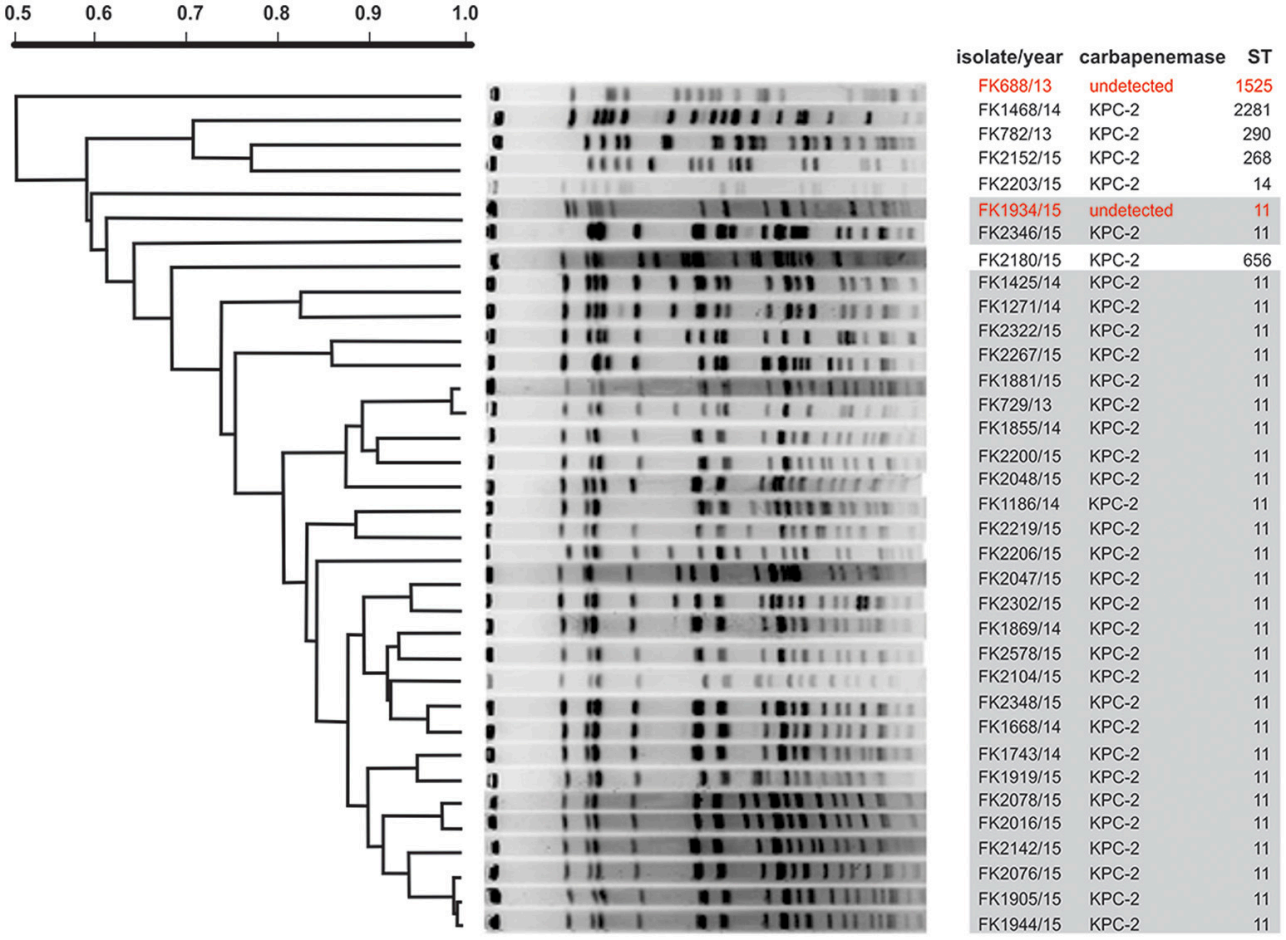
PFGE analysis of XDR *K. pneumoniae* isolates. In order to generate diagnostic genomic DNA fragmentation fingerprints, genomic DNA from each of the XDR *K. pneumoniae* isolates was digested using *XbaI* and subjected to pulsed-field gel electrophoresis. DNA fingerprints were revealed by Gel Red staining. For MLST-based categorization of the strains, the sequences of seven housekeeping genes (i.e., *gapA, infB, mdh, pgi, phoE, rpoB*, and *tonB*) were analyzed, and the PFGE patterns have been organized according to a dendogram of 35 XDR *K. pneumoniae* isolates based on MLST analysis. In red text are those isolates with no detectable carbapenemase genes. The gray box highlights the prevalence of the ST11 sequence type.

Molecular typing enables detection of nosocomial transmission of bacterial pathogens, and can assist in identifying the routes of transmission in hospital settings. Multilocus sequence typing (MLST) identified seven different STs with ST11 being the predominant clone (Table 2, Figure 1). This finding is consistent with the epidemic dissemination of *K. pneumoniae* carbapenemase (KPC)-producing *K. pneumoniae* in China described in another study (Zhou et al., 2015). Another six STs were also identified: ST1525 (FK688/13), ST290 (FK782/13), ST2281 (FK1468/14), ST268 (FK2152/15), ST14 (FK2203/15), ST656 (FK2180/15). The novel ST2281 (allelic profile: 4-1-1-22-7-4-35) which was a multiple locus variant, has not previously been documented and has been submitted to the MLST database.

**Table 2:** Molecular characteristics and epidemiological analysis of XDR *K. pneumoniae* clinical isolates in Wenzhou, 2013–2015 (*n* = 35).

**Isolate/year**	**Resistance determinants**				**Epidemiological analysis**			
	**Carbape-nemases**	**Other β-lactamases genes**	**Quinolone resistance genes**	**Aminoglycoside resistance determinants**	**Size of *bla*_KPC−2_ plasmid (kb)**	**Transcon-jugant**	**Replicon types**	**STs**
FK 688/13	—	*bla*_CMY_, *bla*_DHA−1_	—	AAC(6′)-Ib, APH(3′)-Ia		—	—	1,525
FK 729/13	KPC-2	*bla*_CTX−M−14_, *bla*_SHV−11_	*gyrA* (S83I, D87G), *parC* (S80I)	AAC(6′)-Ib, *rmtB*	—	—	IncFrepB	11
FK 782/13	KPC-2	*bla*_CTX−M−65_, *bla*_SHV−11_	*qnrS*	AAC(6′)-Ib	—	—	—	290
FK 1186/14^a^	KPC-2	*bla*_CTX−M−65_, *bla*_SHV−11_	*qnrS, gyrA* (S83I, D87G), *parC* (S80I)	AAC(6′)-Ib, APH(3′)-Ia, *rmtB*	54.7	J1186	IncFrepB	11
FK 1271/14	KPC-2	—	*gyrA* (S83I, D87G), *parC* (S80I)	AAC(6′)-Ib, *rmtB*	—	—	IncFrepB	11
FK 1425/14	KPC-2	*bla*_CTX−M−14_, *bla*_SHV−11_	*gyrA* (S83I, D87G), *parC* (S80I)	AAC(6′)-Ib, APH(3′)-Ia,	—	—	IncFrepB	11
FK 1468/14^a^	KPC-2	*bla*_CTX−M−14_, *bla*_SHV−11_	*qnrB, gyrA* (S83I, D87G)	AAC(6′)-Ib, AAC(3)-IV	54.7	J1468	—	2,281
FK 1668/14	KPC-2	*bla*_CTX−M−14_, *bla*_SHV−11_	*gyrA* (S83I, D87G), *parC* (S80I)	AAC(6′)-Ib, *rmtB*	—	—	IncFrepB	11
FK 1743/14^a^	KPC-2	*bla*_CTX−M−65_, *bla*_SHV−11_	*gyrA* (S83I, D87G), *parC* (S80I)	AAC(6′)-Ib, *rmtB*	167.1	J1743	IncFrepB	11
FK 1855/14	KPC-2	*bla*_CTX−M−14_, *bla*_SHV−11_	*gyrA* (S83I, D87G), *parC* (S80I)	AAC(6′)-Ib, APH(3′)-Ia, *rmtB*	—	—	IncFrepB	11
FK 1869/14	KPC-2	*bla*_CTX−M−65_, *bla*_SHV−11_	*gyrA* (S83I, D87G), *parC* (S80I)	AAC(6′)-Ib, *rmtB*	—	—	IncFrepB	11
FK 1919/15	KPC-2	*bla*_CTX−M−14_, *bla*_SHV−11_	*aac(6′)-Ib-cr, gyrA* (S83I, D87G), *parC* (S80I)	AAC(6′)-Ib, APH(3′)-Ia, *rmtB*	—	—	IncFrepB	11
FK 2048/15	KPC-2	*bla*_CTX−M−65_, *bla*_SHV−11_	*qnrS, parC* (S80I)	AAC(6′)-Ib	—	—	IncFrepB	11
FK 2104/15	KPC-2	*bla*_CTX−M−65_, *bla*_SHV−11_	*aac(6′)-Ib-cr, gyrA* (S83I, D87G), *parC* (S80I)	AAC(6′)-Ib, *rmtB*	—	—	IncFrepB	11
FK 2152/15^a^	KPC-2	*bla*_CTX−M−15_, *bla*_SHV−11_	*qnrS*	AAC(6′)-Ib	54.7	J2152	—	268
FK 2200/15	KPC-2	*bla*_CTX−M−65_, *bla*_SHV−11_	*gyrA* (S83I, D87G), *parC* (S80I)	AAC(6′)-Ib, APH(3′)-Ia, *rmtB*	—	—	IncFrepB	11
FK 2203/15	KPC-2	*bla*_CTX−M−15_	—	AAC(6′)-Ib	—	—	—	14
FK 2206/15	KPC-2	*bla*_TEM−1_, *bla*_SHV−11_	*gyrA* (S83I, D87G), *parC* (S80I)	AAC(6′)-Ib, *rmtB*	—	—	IncFrepB	11
FK 2219/15	KPC-2	*bla*_CTX−M−14_	*qnrB, gyrA* (S83I, D87G), *parC* (S80I)	AAC(6′)-Ib, APH(3′)-Ia, *rmtB*	—	—	IncFrepB	11
FK 2267/15	KPC-2	*bla*_CTX−M−65_, *bla*_SHV−11_	*gyrA* (S83I, D87G), *parC* (S80I)	AAC(6′)-Ib, APH(3′)-Ia, *rmtB*	—	—	IncFrepB	11
FK 2302/15^a^	KPC-2	*bla*_CTX−M−15_, *bla*_CTX−M−65_, *bla*_TEM−1_, *bla*_SHV−11_	*qnrS, gyrA* (S83I, D87G), *parC* (S80I)	AAC(6′)-Ib, *rmtB*	54.7	J2302	IncFrepB	11
FK 2322/15^a^	KPC-2	*bla*_CTX−M−14_	*gyrA* (S83I, D87G), *parC* (S80I)	AAC(6′)-Ib, *rmtB*	54.7	J2322	—	11
FK 2346/15^a^	KPC-2	*bla*_CTX−M−65_, *bla*_SHV−11_	*gyrA* (S83I, D87G), *parC* (S80I)	AAC(6′)-Ib, *rmtB*	54.7	J2346	IncFrepB	11
FK 2348/15	KPC-2	*bla*_CTX−M−65_, *bla*_SHV−11_	*gyrA* (S83I, D87G), *parC* (S80I)	AAC(6′)-Ib, *rmtB*	—	—	IncFrepB	11
FK 2578/15	KPC-2	*bla*_CTX−M−14_, *bla*_SHV−11_	*gyrA* (S83I, D87G), *parC* (S80I)	AAC(6′)-Ib, *rmtB*	—	—	IncFrepB	11
FK 1881/15^a^	KPC-2	*bla*_CTX−M−65_, *bla*_TEM−1_, *bla*_SHV−11_	*gyrA* (S83I, D87G), *parC* (S80I)	AAC(6′)-Ib, APH(3′)-Ia, *rmtB*	54.7	J1881	IncFrepB	11
FK 1905/15	KPC-2	*bla*_CTX−M−14_, *bla*_TEM−1_, *bla*_SHV−11_	*gyrA* (S83I, D87G), *parC* (S80I)	AAC(6′)-Ib, APH(3′)-Ia, AAC(3)-IV, *rmtB*	—	—	IncFrepB	11
FK 1934/15	—	*bla*_SHV−11_, *bla*_DHA−1_	*aac(6′)-Ib-cr, gyrA* (S83I, D87G), *parC* (S80I)	AAC(6′)-Ib, APH(3′)-Ia, AAC(3)-IV, *rmtB*	—	—	—	11
FK 1944/15	KPC-2	*bla*_CTX−M−65_, *bla*_TEM−1_, *bla*_SHV−11_	*gyrA* (S83I, D87G), *parC* (S80I)	AAC(6′)-Ib, *rmtB*	—	—	IncFrepB	11
FK 2076/15^a^	KPC-2	*bla*_CTX−M−65_, *bla*_TEM−1_	*gyrA* (S83I, D87G), *parC* (S80I)	AAC(6′)-Ib, APH(3′)-Ia, *rmtB*	54.7	J2076	—	11
FK 2016/15	KPC-2	*bla*_CTX−M−14_, *bla*_TEM−1_, *bla*_SHV−11_	*gyrA* (S83I, D87G), *parC* (S80I)	AAC(6′)-Ib, APH(3′)-Ia, *rmtB*	—	—	IncFrepB	11
FK 2047/15^a^	KPC-2	*bla*_CTX−M−65_, *bla*_TEM−1_, *bla*_SHV−11_	*gyrA* (S83I, D87G), *parC* (S80I)	AAC(6')-Ib, APH(3′)-Ia, AAC(3)-IV, *rmtB*	54.7	J2047	IncFrepB	11
FK 2078/15	KPC-2	*bla*_CTX−M−65_, *bla*_TEM−1_	*gyrA* (S83I, D87G), *parC* (S80I)	AAC(6′)-Ib, APH(3′)-Ia, *rmtB*	—	—	—	11
FK 2142/15^a^	KPC-2	*bla*_CTX−M−65_, *bla*_TEM−1_, *bla*_SHV−11_	*gyrA* (S83I, D87G), *parC* (S80I)	AAC(6′)-Ib, APH(3′)-Ia, *rmtB*	54.7	J2142	IncFrepB	11
FK 2180/15^a^	KPC-2	*bla*_CTX−M−14_, *bla*_TEM−1_, *bla*_SHV−11_	*qnrB, aac(6′)-Ib-cr, gyrA* (S83I, D87G), *parC* (S80I)	AAC(6′)-Ib, APH(3′)-Ia, *rmtB*	54.7	J2180	—	656

a *Strains that have successfully transferred carbapenem resistance genes bla_KPC_ to E. coli J53; “—” means undetected*.

The PFGE patterns of the 35 XDR *K. pneumoniae* isolates show a major cluster of 23 closely related isolates that exhibited >80% similarity: these are all KPC-2-producing resistant strains and are all ST11 clones (Figure 1). The rest 12 isolates which exhibited <80% similarity were assigned to seven STs (ST11, ST1525, ST290, ST2281, ST268, ST14, and ST656). Eighteen XDR *K. pneumoniae* isolated from ICU patients (directly admitted and transferred) belong to the predominant cluster, and the other five isolates belonging to the predominant cluster were separated from other departments. A worrying finding is that the close relatedness among the 23 XDR *K. pneumoniae* isolates suggests a phenomenon of clone dissemination within the clinic and the transfer of patients in different medical departments may accelerate clonal spread of pathogens.

### Susceptibilities of XDR *K. pneumoniae*

MIC assays of the patient isolates revealed that each of the XDR *K. pneumoniae* strains were highly resistant to at least one of the β-lactams, carbapenems, aminoglycosides, quinolones, β-lactam/inhibitor combinations, and other clinical antimicrobial agents (Table 3). Consistent with the treatment success is the observation that tigecycline exhibited superior bacteriostasis activity *in vitro*. In addition, 33 isolates were susceptible to polymyxin B (Table 3).

**Table 3:** Antibiotic susceptibility of XDR *K. pneumoniae* isolates.

**Antibiotics**	**MIC (mg/L)**			**Percentage**
	**MIC_50_**	**MIC_90_**	**Range**	**Susceptible/Non-susceptible**
β**-lactams**				
CAZ	128	>128	64 to >128	0/100
CTX	>128	>128	32 to >128	0/100
β**-lactam/Inhibitor**				
SAM	64	>128	64 to >128	0/100
TZP	>128	>128	64 to >128	0/100
**Carbapenems**				
IPM	64	64	8 to >64	0/100
MEM	>64	>64	1 to >64	2.9/97.1
ETP	>64	>64	4 to >64	0/100
**Aminoglycosides**				
AMK	>256	>256	1 to >256	5.7/94.3
GEN	>256	>256	8 to >256	0/100
TOB	256	>256	2 to >256	5.7/94.3
**Quinolones**				
CIP	32	64	1 to 128	2.9/97.1
LEV	16	64	4 to 64	0/100
**Other**				
FOS	>512	>512	64 to >512	0/100
PB	1	1	0.25 to 128	94.3/5.7
TGC	0.5	1	0.25 to 1	100/0

*CAZ, ceftazidime; CTX, cefotaxime; SAM, ampicillin/sulbactam; TZP, piperacillin/tazobactam; IPM, imipenem; MEM, meropenem; ETP, ertapenem; AMK, amikacin; GEN, gentamicin; TOB, tobramycin; CIP, ciprofloxaxin; LEV, levofloxacin; FOS, fosfomycin; PB, polymyxin B; TGC, tigecycline*.

### Distribution of resistance determinants

Diverse resistance determinants were detected among the 35 XDR *K. pneumoniae* strains (Table 2). Of these, 33 strains (94.3%) were detected as co-harboring three or more resistance determinants. There is a global spread of KPC-2 carbapenemase (Naas et al., 2008; Nordmann et al., 2009) that usually explains such findings. However, two strains (FK688 and FK1934) did not express KPC-2 carbapenemase (highlighted in Figure 1). Other β-lactamases were also prevalent, including CTX-M-type ESBLs (31 isolates), SHV (28 isolates), and TEM (11 isolates). In addition, several strains harbored the β-lactamases CMY and DHA-1.

Additional genes conferring drug resistance were sequenced, including the DNA gyrase encoding *gyrA* and the DNA topoisomerase IV encoding *parC*. Amino acid substitutions detected in 31 (88.6%) of the strains were *gyrA* (S83I, D87G) and *parC* (S80I). In addition, mutations were detected in quinolone resistance-determining regions (QRDRs). Intriguingly, 29 of the 30 major ST strains (ST11, ST656) among the fluoroquinolone-resistant strains carried the favorable “double serine” mutations in the *gyrA* and *parC* genes plus an additional-energetically less favorable *gyrA* mutation. A single ST11 strain (2048/15) carried just one favorable serine mutation without any less favorable mutations. In contrast, fluoroquinolone-resistant minor clone strains either failed to harbor any *gyrA* or *parC* mutations (ST1525, ST290, ST268, ST14) or harbored one favorable serine mutation together with a less favorable mutation (ST2281) (Table 2). The 16S-rRNA methylase encoding gene *rmtB* was present in 28 (80%) of the strains, a gene that confers resistance to most clinically relevant aminoglycosides. The plasmid-mediated quinolone resistance genes *qnrB, qnrS*, and *aac(6*′*)-Ib-cr* were also determined as present in 11 (31.4%) of the strains. 35 (100%) of the strains possessed AMEs, including AAC(6′)-Ib (100%), APH(3′)-Ia (48.6%), AAC(3)-IV (11.4%), ANT(2″)-Ia (0%). Taken together, these results demonstrate carriage of multiple genetic determinants for drug resistance in the BSI strains.

### Transferability of plasmids carrying resistance determinants

**Figure 2:**
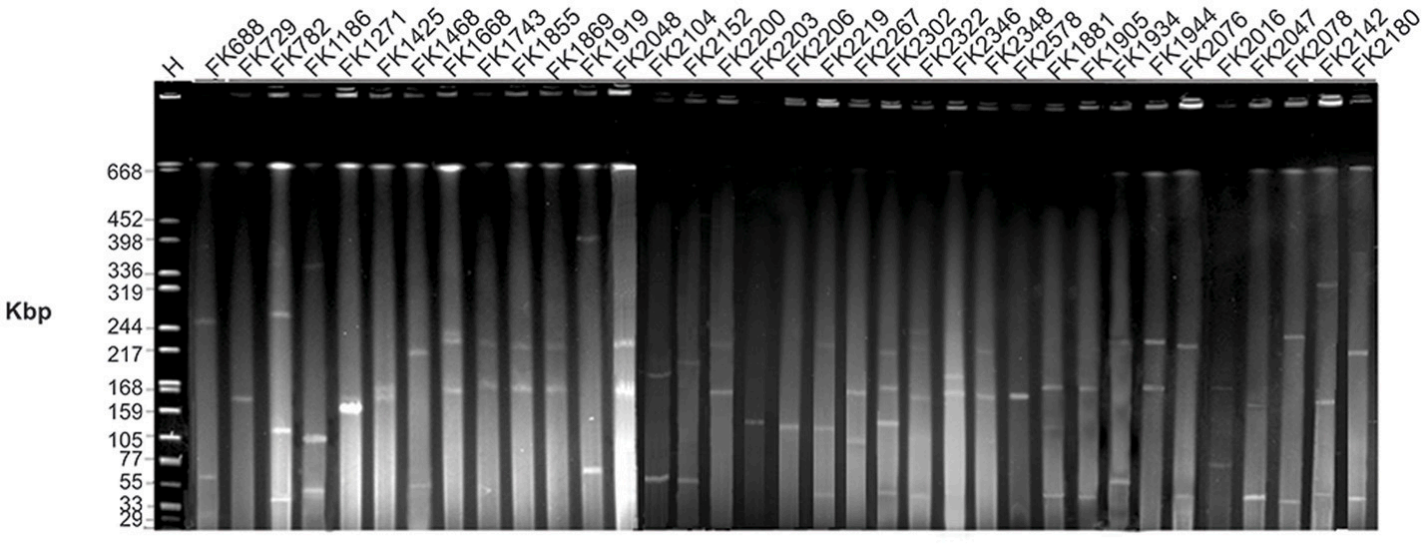
Gel image of S1 PFGE result of all XDR *K. pneumoniae* isolates. Isolates were digested using S1 nuclease and subjected to pulsed-field gel electrophoresis. The gel was subjected to Gel Red staining and analyzed in a CHEF-Mapper XA PFGE system. *H* = size marker strain *Salmonella enterica* ser. Braenderup H9812 digested with *Xba*I.

**Figure 3:**
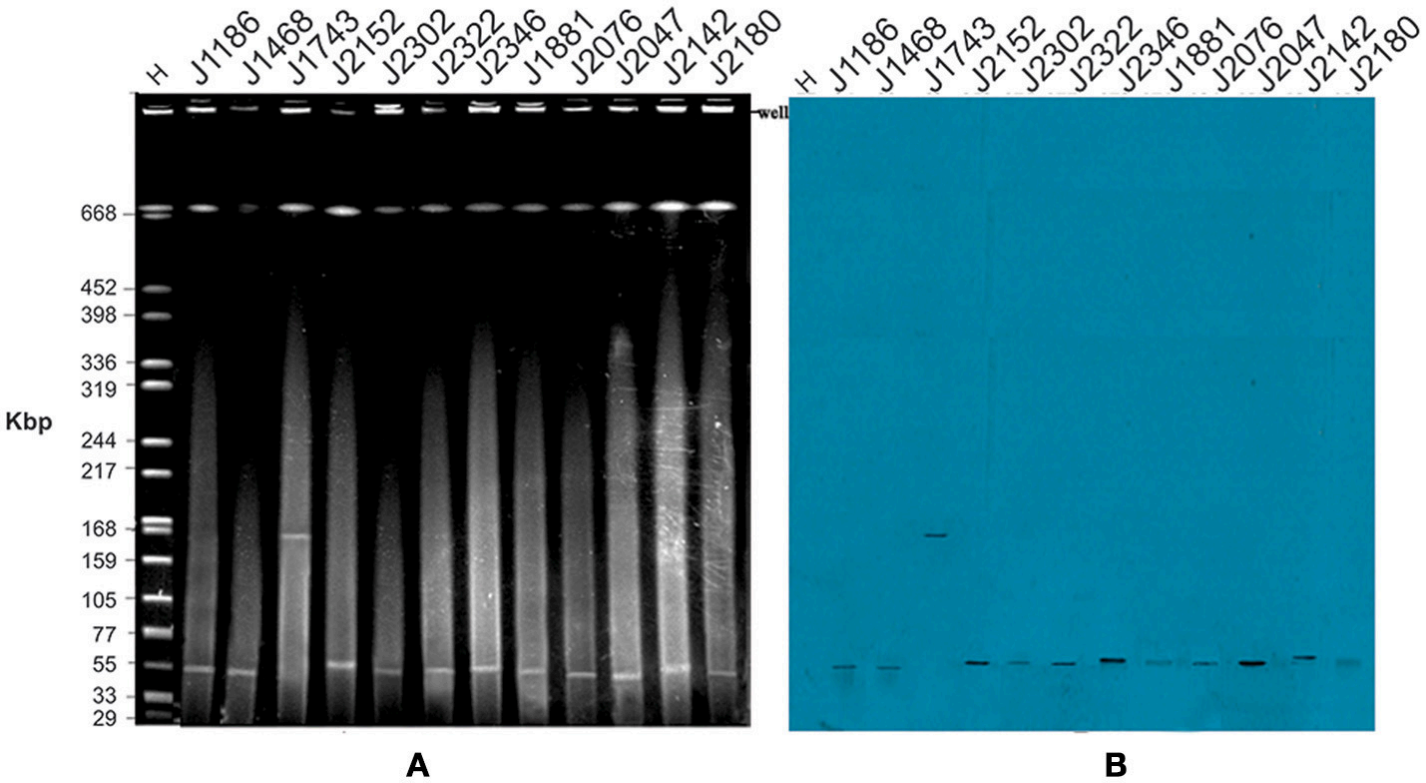
Plasmid profiles of *E. coli* transconjugants. **(A)** In order to verify plasmids carried by the XDR *K. pneumoniae* isolates, transconjugation experiments were established using *E. coli* J53 as a recipient strain. Genomic DNA was isolated from the transconjugants, digested using S1 nuclease and subjected to pulsed-field gel electrophoresis. *H* = size marker strain *Salmonella enterica* ser. Braenderup H9812 digested with *Xba*I; Lanes 2–12 represent transconjugants of the corresponding number of donor isolates, all of the isolates code consistent with Table 3. **(B)** The corresponding Southern blot, hybridized with a DNA probe to the *bla*_KPC−2_ sequence.

KPC-2 and other resistance determinants are often carried on plasmids (Kuai et al., 2014), and S1-PFGE revealed the presence of one or more plasmids in each of the isolated strains (Figure 2). The detected plasmids by S1-PFGE ranged in size from ~50 to ~390 kb. For an initial assessment of the plasmids, transconjugation experiments were established using *E. coli* J53 as the recipient. Carbapenemase genes from 12 strains were successfully transferred to the recipient *E. coli* J53, but no transconjugants were recovered for the other 23 donor strains, despite repeated attempts, may due to the lack of sex factor F or conjugative plasmids which are key vehicles and contain simultaneously important elements, including an origin of transfer, DNA-processing factors (a relaxase and accessory proteins), and mating pair formation proteins (Achtman et al., 1971; Goessweiner-Mohr et al., 2014) (Table 2). The MIC values for the transconjugants determined for β-lactam antibiotics were consistent with the transfer of carbapenemase expression. Southern blot analysis revealed the presence of the *bla*_KPC−2_ in the transconjugants and in 11 cases corresponded to a plasmid of ~50 kb (Figure 3), while strain J1743 carried the *bla*_KPC−2_ gene on a plasmid of ~160 kb (Figure 3). These results are in accordance with the S1-PFGE analysis of the isolates (Figure 2). Some of these strains were found to be F*repB*-positive by PBRT, showing that these plasmids belong to an IncF-type.

### SDS-Page of outer membrane proteins

**Figure 4:**
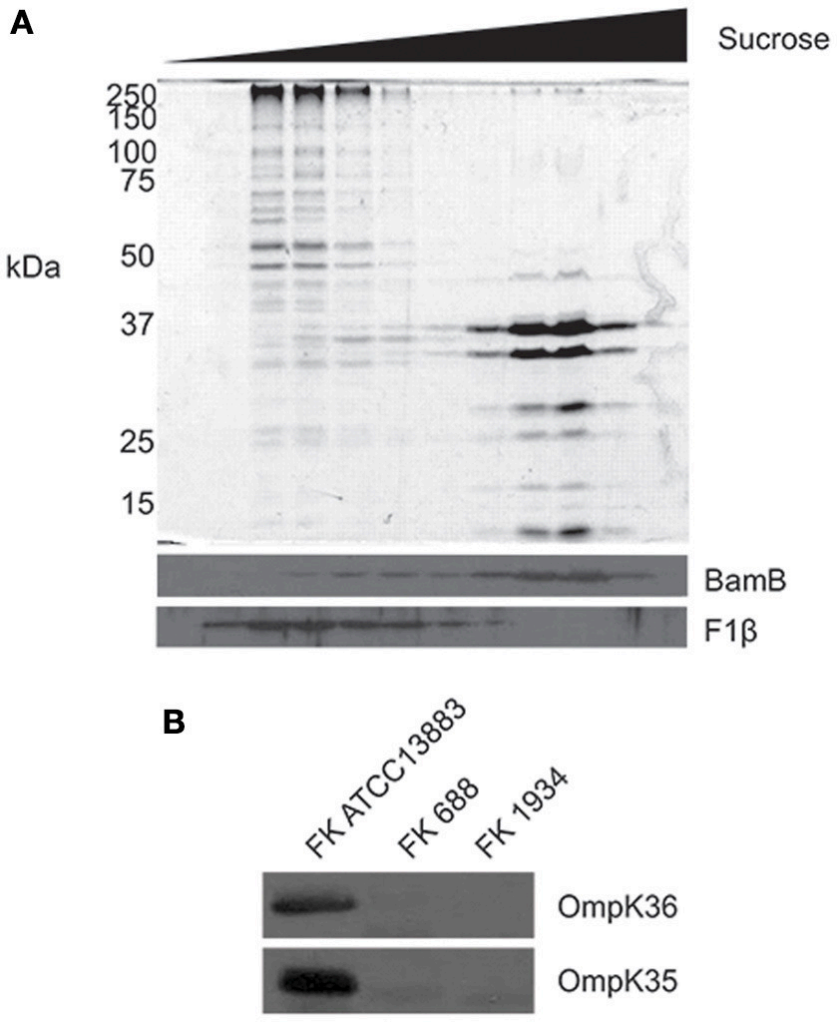
Remodeling of the outer membrane proteome in the XDR *K. pneumoniae* isolates. **(A)** Total membranes were isolated from *K. pneumoniae* ATCC 13883 and subjected to sucrose density fractionation. Membrane fractions were subject to SDS-PAGE, and then Coomassie blue staining (top panel) and immunoblotting (bottom panel) using antisera recognizing known outer (BamB) and inner (F1β) membrane proteins. **(B)** Whole cell lysates from *K. pneumoniae* ATCC 13883 and *K. pneumoniae* clinical isolates FK688 and FK1934 were analyzed by SDS-PAGE and western immunoblotting for OmpK35 and OmpK36.

The major porins of *E. coli*, OmpF and OmpC, provide for most of the flux of molecules up to ~650 Da across the outer membrane (Nikaido et al., 1983; Sugawara et al., 2016) and thereby play a role in the susceptibility of *E. coli* to antibiotics (Nikaido, 2003; Zgurskaya et al., 2015). The homologous porins of *K. pneumoniae*, OmpK35 and OmpK36, have been highlighted as the primary channels through which beta-lactams cross the outer membrane (Kaczmarek et al., 2006). OmpK35 and OmpK36 are highly abundant proteins of the outer membrane in *K. pneumoniae*, and can be readily detected by Coomassie blue staining after SDS-PAGE analysis of the outer membranes (Rath et al., 2009). A procedure was optimized to purify outer membranes from the control strain *K. pneumoniae* ATCC 13883, using sucrose gradient fractionation to separate outer membranes from inner membranes (Figure 4A). Immunoblotting for marker proteins F_1_β and BamB served as controls for the fractions (Figure 4A). Comparative analysis of outer membrane fractions isolated from the carbapenem-resistant, yet carbapenemase-negative strains FK688 and FK1934 using antibodies specific for OmpK35 and OmpK36 showed no detectable expression of OmpK35 and OmpK36 (Figure 4B). PCR primers designed to amplify regions of the corresponding loci failed to detect *ompK35* and *ompK36* in FK688 and FK1934 (data not shown).

## Discussion

Between 2013 and 2015, the incidence rate of all nosocomial infections in this major public hospital in southern China was 2.23%, comparable to other grade A tertiary hospitals that range from 2 to 4% (Mu et al., 2015; Zhao, 2015). The current study was motivated by the observation that bloodstream infection became a considerable cause of mortality in nosocomial infections accounting for ~14%, ranking immediately after respiratory tract infections and incision infections in ICUs (Russotto et al., 2015). In this period from 2013 to 2015, *K. pneumoniae* was frequently isolated from these BSIs patients, and predominantly with a multiple drug resistant phenotypes. It is a growing global reality, that XDR *K. pneumoniae* nosocomial infections are associated with delays in appropriate therapy, and therefore pose serious challenges to clinically effective therapeutic options worldwide (Lim et al., 2015). The increasing prevalence and global dissemination of these clinical XDR strains gravely threatens public health (Zhao et al., 2015).

While our study has been conducted in a single medical center, the samples were sourced from the First Affiliated Hospital of Wenzhou Medical University, which is one of the largest comprehensive hospitals integrating medical care, clinical practice teaching and scientific research. The hospitals outpatient clinics service close to five million patients per annum, taking responsibility for the medical care of a 30 million population in southern Zhejiang and eastern Fujian areas. Environments such as this provide a valuable resource to track new developments in hospital acquired infections and monitor the dissemination of new resistance genes for ongoing studies.

Respiratory infections could be the key source of XDR *K. pneumoniae* BSIs, which require a prolonged antibiotic therapy and are considered as an important factor for acquired resistance of pathogens (Ryan et al., 2015). The case-fatality rate attributed to BSIs caused by XDR *K. pneumoniae* here was 40% (14/35), the higher than the overall mortality rate (15–20%) associated with BSIs in previous studies (Russotto et al., 2015). The high ICU admission rate (*n* = 21, 60%) that was observed in the present study, may relate to hypoimmunity resulting from severe comorbidities and invasive care procedures (Woodford et al., 2004; Bouza et al., 2013; Martelius et al., 2016). The clinical retrospective data showed that 57% of cases received broad-spectrum antibiotics, including third-generation cephalosporin, carbapenems, and tigecycline. Recent studies have shown that carbapenems are generally considered as part of combination therapy when carbapenem MICs are <8 mg/L (Daikos et al., 2014), and this had become a standard treatment regime. However, 34 of the 35 isolates of XDR *K. pneumoniae* in this study were non-susceptible to meropenem and, even in combination therapy, meropenem was not protective against mortality of BSIs patients (*P* = 0.015). Thus, rapid typing and identification of specific BSIs phenotypes is essential to avoid unnecessary and inappropriate antimicrobial regimens. Our data showed that an alternative treatment regime of tigecycline in combination with other antimicrobial agents was significantly effective (*P* = 0.016).

The emergence and spread of XDR *K. pneumoniae* over the last decade is a major, global concern (Naparstek et al., 2012), and the findings of this specific study are relevant to problems faced by clinicians around the globe. In the last several years, antibiotic resistance mediated by plasmids has been increasing at a remarkable rate (Yang et al., 2011), especially through genes encoding carbapenemase, PMQRs and 16S-RMTase. Strikingly, most of the isolates in this study possessed KPC-2 and co-harbored three or more classes of resistance genes. The high prevalence rate of KPC-2 among these isolates exceeded that found in other investigations across the world (Bradford et al., 2004). Previous studies proposed that *bla*_KPC−2_ resides on transmissible plasmids, which frequently co-harbor other resistance elements (Endimiani et al., 2008), leading to MDR and XDR pathogens. Even where carbapenemase genes are not evident, carbapenem-resistant phenotypes were observed; while they carried AmpC β-lactamase genes (*bla*_CMY_ and *bla*_DHA−1_), no carbapenemase genes were detected in two strains from this study, FK688 and FK1934. Notably, these two strains were not found to express OmpK35 and OmpK36. We suggest that the absence of the major porins in the bacterial outer membrane reduced permeability to carbapenem, thereby enhancing carbapenem resistance.

Using molecular analysis, we located *bla*_KPC−2_ on ~50 and ~160 kb transferable plasmids which co-harbored *bla*_rmtB_ and *bla*_CTX−M−9_. In consideration of the problems that smaller plasmids may not be detected by S1-PFGE, plasmids of randomly selected 10 strains and 6 transconjugants were extracted and separated by agarose gel electrophoresis. The result revealed the presence of smaller plasmids which were not detected by S1-PFGE in all of the 10 strains and 2 of the 6 transconjugants. The gel was then subject to Southern blot analysis, after transfer to a positively charged nylon membrane by the capillary method. The membrane was subject to hybridization with labeled *bla*_KPC−2_ probes, nevertheless, hybridization signals were only observed in large plasmids (>54.2 kb) (Figure S1). Figure S1 was available in supplementary data, and demonstrate that smaller plasmids didn't mediate the widespread dissemination of resistant genes *bla*_KPC−2_ in Wenzhou. However, investigation of other resistant genes harbored by smaller plasmids are still warranted.

The genetic environment surrounding the *bla*_KPC−2_ gene which was located on various plasmids among the KPC-producing *Enterobacteriaceae* iaolated from China is primarily reported to be the integrin of Tn*3*-based transposon. The truncated *bla*_TEM_ gene fragment is the representative structure of the Tn*3* transposon (Luo et al., 2013). In present study, 6 isolates carrying *bla*_KPC−2_ which located in ~50 kb transferable plasmids were detected to harbor *bla*_TEM_ as well, so we speculate that the *bla*_KPC−2_-surrounding nucleotide sequence contain integron structure of Tn*3*-based transposon.

The predominant *bla*_KPC_ plasmid type was IncF, and was associated with *K. pneumoniae* ST258 (Chen et al., 2013). We speculate that most (7/12) transferable *bla*_CTX−M_ and *bla*_KPC−2_ genes may be located on the IncF plasmid, which is capable of clonal expansion and horizontal dissemination among *Enterobacteriaceae* bacteria (Doumith et al., 2012). Previous studies have shown that individual patients can be positive for IncF plasmids for more than 3 years (Onnberg et al., 2014), which might reflect either the persistence of the pathogen clone, or persistence of the plasmid transmitted to other resident bacteria. ESBLs genes carried by plasmids are also detected in various lineages of the *Enterobacteriaceae* (Tham et al., 2012).

Since multiple resistant determinants rendering almost all antimicrobials ineffective and poor survival in patients infected with XDR *K. pneumoniae*, tigecycline and polymyxin B seemed to be the optional choices. Notably, multiple amino acid substitutions, including both *gyrA* (S83I, D87G) and *parC* (S80I) were observed among ST11 strains which was predominant clone except a single ST11 strain (2048/15), being apparently more than that detected in minor clone strains. Resistance mutations decreased the affinity of gyrase and topoisomerase IV for fluoroquinolones, and our findings also illustrate that favorable mutations and consequently superior fitness, closely associated with high-level resistance to fluoroquinolones, could contribute to the promotion of the major international high-risk clone (ST11) of multidrug-resistant *K. pneumoniae*. These findings are in complete agreement with those published by Tóth et al. (2014).

KPC-2-producing *K. pneumoniae* isolates have been reported worldwide, and the dominant KPC-producing clone ST258 is implicated in more than 70% of reported outbreaks in America (Kitchel et al., 2009). Other STs (ST1525, ST290, ST14, ST268, ST656, etc.) have not been reported to be MDR/XDR global clones. The predominant clone now is ST11, which is a variant of the pandemic ST258 clone and played an important role in the epidemic dissemination of *bla*_KPC−2_ (Cuzon et al., 2010). High homology of KPC-2-producing *K. pneumoniae* ST11 has been observed by PFGE, which indicated clonal dissemination in our hospital in recent years, reinforcing the viewpoint speculated by Qi et al. that ST11 *K. pneumoniae* could be considered as a plasmid scavenger that amplifies plasmid dissemination (Qi et al., 2011).

Smaller plasmids can be important resistance reservoirs harboring multiple determinants, and many of them are high copy number conferring the host bacterial strains high levels of resistance. Therefore, comprehensive analysis and study of smaller plasmids are essential for understanding the role of smaller plasmids play in developing of XDR strains. In addition, studying biology of virulence of XDR isolates, and gene environment of the plasmids (~50 and ~160 kb) harboring *bla*_KPC−2_ are worthwhile, and the lack of corresponding researches are deficiencies of present study, further explorations are still warranted in follow-up studies.

## Conclusion

In summary, the complex phenotypes of these XDR *K. pneumoniae* strains were associated with multiple resistance determinants and remodeling of the outer membrane proteome. In addition, favorable gyrase and topoisomerase IV mutations and consequently superior fitness contributed to the promotion of the predominant clone (ST11) of multidrug-resistant *K. pneumoniae* in Wenzhou, China. The dissemination and epidemicity of clinical XDR *K. pneumoniae* strains result from horizontal transmission of multiple resistance determinants via IncF plasmids. KPC-2-producing *K. pneumoniae* ST11 played a crucial role. The clonal dissemination of XDR ST11 demonstrated that efficient screening, intensive surveillance, strict disinfection procedure and prompt quarantine measures are urgently needed to restrain the emergence and transmission of XDR strains in hospital settings. The high mortality attributed to BSIs caused by XDR *K. pneumoniae* is an alert to clinicians to establish rational and effective combination drug therapy.

## Author contributions

TZ, BL, and JC contributed to the design of the experiments. WB, HL, RD, BL, VT, and LC performed the experiment. WB, HL, and BL wrote the initial draft of the manuscript. TZ, TL, WB, HL, RD, BL, VT, JC, LC, JW, and RS contributed to the acquisition, analysis, interpretation of the data included in this manuscript. TZ, TL, WB, HL, BL, JC, LC, and RS revised the manuscript. All authors approves of the final manuscript being submitted and agree to be accountable for the work detailed in the submitted manuscript.

### Conflict of interest statement

The authors declare that the research was conducted in the absence of any commercial or financial relationships that could be construed as a potential conflict of interest.
