# Supplementary material for: Extensively Drug-Resistant Klebsiella pneumoniae Causing Nosocomial Bloodstream Infections in China: Molecular Investigation of Antibiotic Resistance Determinants, Informing Therapy, and Clinical Outcomes
Source: Front Microbiol. 2017 Jun 30;8:1230. doi: 10.3389/fmicb.2017.01230 (PMC5492486; doi:10.3389/fmicb.2017.01230)
Supplement: Supplementary file 1 [file Presentation1.PDF]

Smaller plasmid profiles and Southern blot hybridization of the randomly selected 10 XDR *K. pneumonia* isolates and 6 transconjugants. (A) Plasmid DNA extracted from ten XDR *K. pneumonia* isolates and six transconjugants was separated by agarose gel electrophoresis. Lanes 1-10 represent FK729, FK1869, FK1934, FK2047, FK2078, FK2206, FK2267, FK2302, FK2348, FK2578. Lanes 11 to 16 represent J1186, J1881, J1743, J2076, J2142, J2180; *E. coli* V517 served as the marker. (B) Southern blot of the gel shown in (A) with the labelled $bla_{\text{KPC-2}}$ probe.

Larger plasmids ($\geq$ 54.2kb) could not be separated by agarose gel electrophoresis and located aggregately in the plasmids (~54.2kb). The result revealed that hybridization signals were only observed in large plasmids ($\geq$54.2 kb).
