# Supplementary figures and images for: Extensively Drug-Resistant Klebsiella pneumoniae Causing Nosocomial Bloodstream Infections in China: Molecular Investigation of Antibiotic Resistance Determinants, Informing Therapy, and Clinical Outcomes
Source: Front Microbiol. 2017 Jun 30;8:1230. doi: 10.3389/fmicb.2017.01230 (PMC5492486; doi:10.3389/fmicb.2017.01230)

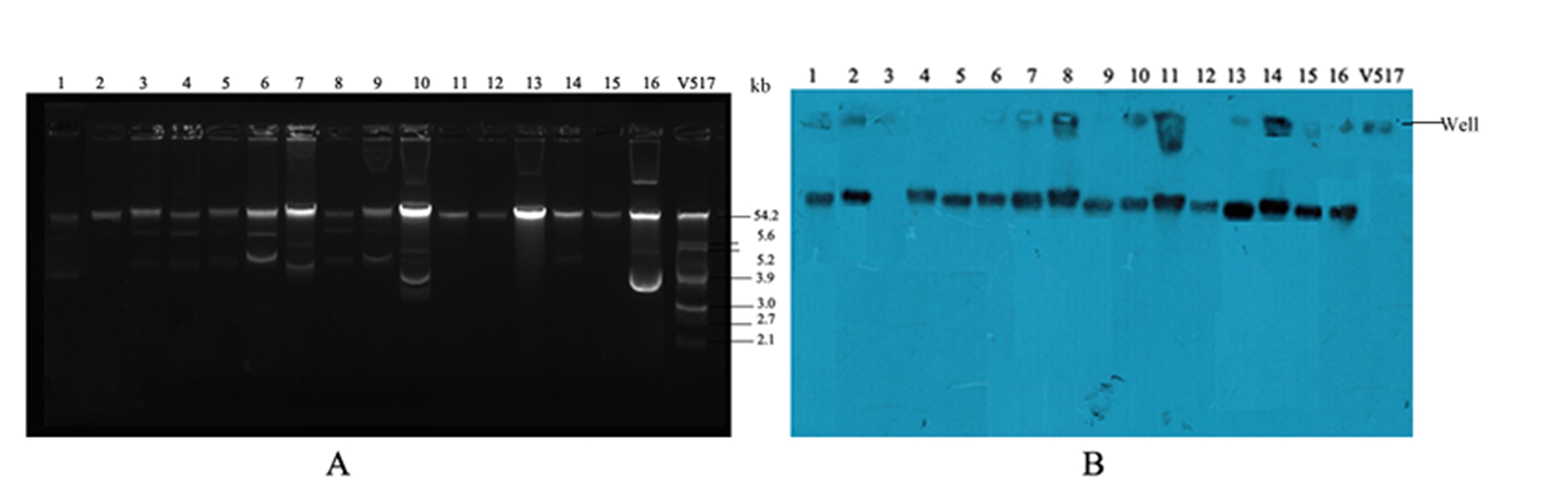

Supplement: Supplementary file 2 [file Image1.TIF]
